# HDAC is indispensable for IFN-γ-induced B7-H1 expression in gastric cancer

**DOI:** 10.1186/s13148-018-0589-6

**Published:** 2018-12-11

**Authors:** Rui Deng, Peng Zhang, Weizhen Liu, Xiangyu Zeng, Xianxiong Ma, Liang Shi, Tao Wang, Yuping Yin, Weilong Chang, Pei Zhang, Guobin Wang, Kaixiong Tao

**Affiliations:** 1 Department of Gastrointestinal Surgery, Union Hospital, Tongji Medical College, Huazhong University of Science and Technology, Wuhan, 430022 China; 2 Department of General Surgery, The First Affiliated Hospital of Zhengzhou University, Zhengzhou, 450000 China

**Keywords:** Gastric cancer, Immune evasion, B7-H1, HDAC, IFN-γ

## Abstract

**Background:**

B7 homolog 1 (B7-H1) overexpression on tumor cells is an important mechanism of immune evasion in gastric cancer (GC). Elucidation of the regulation of B7-H1 expression is urgently required to guide B7-H1-targeted cancer therapy. Interferon gamma (IFN-γ) is thought to be the main driving force behind B7-H1 expression, and epigenetic factors including histone acetylation are recently linked to the process. Here, we investigated the potential role of histone deacetylase (HDAC) in IFN-γ-induced B7-H1 expression in GC. The effect of Vorinostat (SAHA), a small molecular inhibitor of HDAC, on tumor growth and B7-H1 expression in a mouse GC model was also evaluated.

**Results:**

RNA-seq data from The Cancer Genome Atlas revealed that expression of B7-H1, HDAC1–3, 6–8, and 10 and SIRT1, 3, 5, and 6 was higher, and expression of HDAC5 and SIRT4 was lower in GC compared to that in normal gastric tissues; that HDAC3 and HDAC1 expression level significantly correlated with B7-H1 in GC with a respective *r* value of 0.42 (*p* < 0.001) and 0.21 (*p* < 0.001). HDAC inhibitor (Trichostatin A, SAHA, and sodium butyrate) pretreatment suppressed IFN-γ-induced B7-H1 expression on HGC-27 cells. HDAC1 and HDAC3 gene knockdown had the same effect. SAHA pretreatment or HDAC knockdown resulted in impaired IFN-γ signaling, demonstrated by the reduction of JAK2, p-JAK1, p-JAK2, and p-STAT1 expression and inefficient STAT1 nuclear translocation. Furthermore, SAHA pretreatment compromised IFN-γ-induced upregulation of histone H3 lysine 9 acetylation level in B7-H1 gene promoter. In the grafted mouse GC model, SAHA treatment suppressed tumor growth, inhibited B7-H1 expression, and elevated the percentage of tumor-infiltrating CD8+ T cells.

**Conclusion:**

HDAC is indispensable for IFN-γ-induced B7-H1 in GC. The study suggests the possibility of targeting B7-H1 using small molecular HDAC inhibitors for cancer treatment.

**Electronic supplementary material:**

The online version of this article (10.1186/s13148-018-0589-6) contains supplementary material, which is available to authorized users.

## Background

Gastric cancer (GC) is one of the most prevalent health threats worldwide, ranking fifth for cancer incidence and third for cancer deaths, with the highest incidence and morbidity occurring in Eastern Asian countries including China, Japan, and South Korea [1, 2]. A hallmark of GC is its escape from immunological surveillance and evasion from immunological targeting [3, 4]. One of the important mechanisms of anti-tumor immunity evasion is that tumor cells overexpress B7 homolog 1 (B7-H1), a checkpoint molecule, which can bind to programmed cell death protein 1 (PD-1) on activated T cell surface to induce T cell apoptosis, anergy, and exhaustion [5–8]. A recent meta-analysis further showed that B7-H1 expression on tumor cells was associated with a poor prognosis of GC patients, indicating a role of B7-H1 in promoting GC progression [9]. The B7-H1 blockade has been demonstrated to be a promising treatment strategy for advanced stage cancer in clinical trials. Anti-B7-H1 monoclonal antibodies elicited durable tumor remission and prolonged survival of selected patients with a broad spectrum of cancer, such as urothelial carcinoma and lung cancer [10–12].

The exact force driving tumor B7-H1 overexpression remains to be determined. Cytokines, including interferon gamma (IFN-γ), tumor necrosis factor alpha (TNF-α), and interleukin 2 (IL-2), as well as dysregulated oncogenic signaling involving mitogen-activated protein kinases (MAPK) and epidermal growth factor receptor (EGFR), are all reported to be contributing factors in vitro, among which IFN-γ is the strongest inducer [13–17]. Based on the close inspection of melanoma specimens, noting that B7-H1 expression was restricted to the colocalized area of tumor cell and lymphocytes, the adaptive resistance mechanism was proposed. In a reciprocal way, IFN-γ secreted by activated effector T cells encountering tumor cells induces a strong expression of B7-H1 on the latter [18].

Epigenetic factors played a role in B7-H1 expression. Trimethylation of lysine 4 on histone H3 (H3K4me3) was enriched in the B7-H1 promoter in pancreatic tumor cells to activate B7-H1 transcription [19]. JQ-1, a specific inhibitor which blocked bromodomain-containing protein 4 (BRD4) binding to the acetylated histones, suppressed the expression of B7-H1, suggesting that histone acetylation took part in B7-H1 regulation [20, 21]. As is known to us, histone acetylation is the result of dynamic equilibrium of histone deacetylase (HDAC) and histone acetyltransferase (HAT) [22]. Of our interest, HDAC is a potential target of therapy in many cancers, and certain HDAC inhibitors (HDACIs) have been applied clinically [23]. Furthermore, HDACIs were shown to have widespread immuno-regulatory functions other than direct cytotoxic effects [24–26].

Herein, we investigated the role of HDAC in IFN-γ-induced B7-H1 expression in GC. We demonstrated that HDAC is indispensable for IFN-γ-induced B7-H1 expression. HDACI impaired IFN-γ signaling and B7-H1 expression. Furthermore, in the mouse model of subcutaneously grafted GC, HDACI treatment downregulated tumor B7-H1 and suppressed tumor growth. Our findings showed that HDAC inhibition is a possible way of targeting B7-H1 in GC.

## Methods

### Bioinformatics analysis

Differential expression of B7-H1, HDAC1–11 and SIRT1–7 between normal and GC tissues was evaluated using public available RNA-seq data from The Cancer Genome Atlas (TCGA) database through UALCAN portal [27, 28]. Correlation analysis between B7-H1, HDAC-11, and SIRT1–7 expression in GC tissues was performed through GEPIA web server using RNA-seq data from TCGA [29, 30]. Acetylation level of histone H3 lysine 27 (H3K27) in CD274 promoter region on seven cell lines was visualized through UCSC Genome Browser using ChIP-seq data from ENCODE [31–33].

### Tissue samples

GC and adjacent tissues were obtained from Union Hospital, Tongji Medical College, Huazhong University of Science and Technology.

### Cell culture and treatment

Human GC cell line HGC27 was purchased from Shanghai Cell Bank of Chinese Academy of Science, China. Mouse GC cell line MFC was purchased from Shanghai Zhong Qiao Xin Zhou Biotechnology Corporation, China. Both cells were cultured with RPMI 1640 (Hyclone, USA) supplemented with 10% fetal bovine serum (Gibco, USA), penicillin (100 u/ml), and streptomycin (100 mg/ml) in a humidified condition at 37 °C. Cells were treated with IFN-γ (Peprotech, USA) at different concentrations for indicated duration of time, alone or following the pretreatment of small molecular inhibitors for 8 h. HDACIs used included Vorinostat (SAHA), sodium butyrate, and Trichostatin A (TSA). GLPG0634, fedratinib, fludarabine, and NSC 74589 were used to selectively inhibit Janus kinase 1 (JAK1), JAK2, and signal transducer and activator of transcription 1 (STAT1) and STAT3, respectively. The small molecular inhibitors are all purchased from Apexbio, USA, and were dissolved in DMSO in storage concentration except that sodium butyrate was in the water.

### Quantitative RT-PCR

Tissue or cultured cells were lysed using RNAiso plus (Takara, Japan), and total RNA was extracted according to the manufacturer’s instructions. cDNA was synthesized using kit RR036 according to the manufacturer’s instructions (Takara, Japan). qPCR was performed on StepOnePlus™ system using kit RR820 according to the manufacturer’s instructions (Takara, Japan). The following primers were used: human B7-H1 forward: TGGCATTTGCTGAACGCATTT, reverse: TGCAGCCAGGTCTAATTGTTTT; human glyceraldehyde-3-phosphate dehydrogenase (GAPDH) forward: CTGGGCTACACTGAGCACC, reverse: AAGTGGTCGTTGAGGGCAATG; human HDAC1 forward: CTACTACGACGGGGATGTTGG, reverse: GAGTCATGCGGATTCGGTGAG; human HDAC2 forward: ATGGCGTACAGTCAAGGAGG, reverse: TGCGGATTCTATGAGGCTTCA; human HDAC3 forward: CCTGGCATTGACCCATAGCC, reverse: CTCTTGGTGAAGCCTTGCATA; and human JAK2 forward: TCTGGGGAGTATGTTGCAGAA, reverse: AGACATGGTTGGGTGGATACC.

### Flow cytometry

For cultured cells, they were trypsin digested and collected after indicated treatment. For resected tumor tissues, they were cut into small pieces and then digested in collagenase I (0.3 mg/ml) at 37 °C for 1 h. The mixture was filtered through a 100-μm strainer to remove undigested tissue blocks. Wash the collected cells using phosphate buffer saline (PBS) containing 1% bovine serum albumin (BSA) (wash buffer) twice. After the final wash, add wash buffer to resuspend cells. Incubate about 1 × 10^6^ cells with 3–5 μl antibody in 100 μl wash buffer at the 4 °C in darkness for 30 min. Wash the cells twice, and perform the analysis on the flow cytometer (BD, USA). The anti-human B7-H1-APC antibody and anti-mouse B7-H1-FITC antibody were purchased from Biolegend, USA.

### Western blot

Tissues or cultured cells were lysed on ice using RIPA to extract total protein. After protein concentration determination using the BCA method, total protein was mixed with loading buffer and boiled at 100 °C for 5 min. Sodium dodecyl sulfate-polyacrylamide gel electrophoresis (SDS-PAGE) was performed according to the standard procedures, followed by protein transfer to PVDF membrane. After blocking, the PVDF membrane was incubated in diluted antibody solution at 4 °C overnight. After wash, the membrane was incubated with secondary horseradish peroxidase (HRP)-conjugated antibody at room temperature for 1 h. The membrane was then subjected to chemiluminescence, and the signal was detected by ChemiDoc MP system (BioRad, USA). Quantification was performed using ImageJ. The antibodies for human B7-H1 and GAPDH were purchased from Abcam, USA, and the antibodies for human HDAC1, HDAC2, HDAC3, JAK1, JAK2, STAT1, phosphorylated JAK1 (p-JAK1), p-JAK2, and p-STAT1 were purchased from Cell Signaling Technology, USA.

### RNA interference

Small interference RNA (siRNA) targeting human HDAC1, HDAC2, HDAC3, and JAK2 gene were synthesized. The target sequences were as follows: HDAC1: GCCGGTCATGTCCAAAGTAAT; HDAC2: GCTGTGAAGTTAAACCGACAA; HDAC3: GCTTCACCAAGAGTCTTAA; and JAK2: AACTCTATCAGCTACAAGACA. siRNA targeting no specific genes were used as negative control (NC). Cells were cultured in six well plates and were transfected with specific siRNA using Lipo6000 reagent (Beyond, China) according to the manufacturer’s instructions. Thirty-six hours later, cells were harvested and target gene expression was determined using western blot.

### Immunofluorescence

Cells growing on cover glasses were fixed using 4% paraformaldehyde. After an immerse wash in PBS, the cell membranes were permeabilized using 0.5% Triton X-100. After blocking with normal goat serum, the cells were incubated with diluted antibody solution at 4 °C overnight. After a wash in PBS, the cells were incubated with diluted secondary PE/CY3-conjugated antibody. After wash, the cells were stained with DAPI. Analyze the signal under a fluorescence microscope (Olympus, Japan).

### Chromatin immunoprecipitation

Chromatin immunoprecipitation (ChIP) was performed using a kit purchased from CST (cat. #9002) according to the manufacturer’s instructions. Briefly, to crosslink DNA and proteins, cells were incubated with 1% formaldehyde for 10 min and quenched with the addition of 1.25 M glycine. Cells were washed in PBS and lysed in ChIP lysis buffer. Lysates were digested with the micrococcal nuclease at 37 °C to achieve DNA fragments with a length of approximate 150–900 bp. After the digestion, sonicate the lysate for three sets of 20 s at 20% maximum amplitude to break the nuclear membrane and clarify the lysate by centrifugation. To perform one immunoprecipitation, roughly 100 μl digested lysate from 4 × 10^6^ cells was needed. Add 400 μl ChIP buffer to 100 μl lysate and remove 10 μl diluted lysate as 2% input. For each immunoprecipitation, add 10 μg antibody to the lysate and incubate with rotation at 4 °C overnight. Then, add 30 μl ChIP-grade protein G agarose beads and incubate with rotation at 4°Cfor 2 h. Pellet protein G agarose beads and wash sequentially with low-salt and high-salt wash buffer. Elute DNA from the beads with elution buffer and reverse crosslinks (including the input sample). DNA purification was performed using spin columns. qPCR was performed as mentioned above. Enrichment of DNA for each IP was calculated using percent input method with the following equation:

$$ \mathrm{Percent}\ \mathrm{input}=2\%\times {2}^{\left(\mathrm{C}\left[\mathrm{T}\right]\ 2\%\mathrm{Input}\ \mathrm{sample}-\mathrm{C}\left[\mathrm{T}\right]\ \mathrm{IP}\ \mathrm{sample}\right)} $$

$$ \mathrm{C}\left[\mathrm{T}\right]=\mathrm{threshold}\ \mathrm{cycle}\ \mathrm{of}\ \mathrm{PCR}\ \mathrm{reaction}. $$

The following antibody was used in ChIP: anti-H3K9Ac (CST). The following primers located in the B7-H1 promoter region were used, as designed in a previous study (Table 1) [34].

**Table 1:** Primers located in the B7-H1 gene promoter region

Name	Sequence	Location from TSS (bp)
B7-H1 pro1	Forward: GGCAAATTCCGTTTGCCTCA	− 1766
	Reverse: TCCTCCTAGATGGCCTGGAT	
B7-H1 pro2	Forward: GCTGGGCCCAAACCCTATT	− 1178
	Reverse: TTTGGCAGGAGCATGGAGTT	
B7-H1 pro3	Forward: CTAGAAGTTCAGCGCGGGAT	− 800
	Reverse: GGCCCAAGATGACAGACGAT	
B7-H1 pro4	Forward: ATGGGTCTGCTGCTGACTTT	− 455
	Reverse: GGCGTCCCCCTTTCTGATAA	
B7-H1 pro5	Forward: GGGGGACGCCTTTCTGATAA	− 364
	Reverse: AAGCCAACATCTGAACGCAC	
B7-H1 pro6	Forward: AGGACGGAGGGTCTCTACAC	+ 95
	Reverse: ATTGGCTCTACTGCCCCCTA	
B7-H1 pro7	Forward: GTAGGGAGCGTTGTTCCTCC	+ 162
	Reverse: GTGTAGAGACCCTCCGTCCT	
B7-H1 pro8	Forward: TAGGGGGCAGTAGAGCCAAT	+ 229
	Reverse: CAAAACTGAATCGCGCCTGG	

### In vivo analysis

We constructed a syngeneic, immune competent mouse GC model by injecting MFC cells subcutaneously into 615 mice. MFC cell line was originally isolated from a primary forestomach carcinoma of an inbred 615 strain mouse. Five-week-old female 615 mice were purchased from Tianjin Institute of Hematology. 2 × 10^6^ MFC cells were injected subcutaneously into the right inguinal region. Three days after the injection, 12 mice were randomly assigned to the experimental or control group. For the experimental group, SAHA were injected subcutaneously with a daily dose of 20 mg/kg. For the control group, vehicle 6 μl DMSO + 30 μl PEG 300 + 70 μl Tween 80 was used. Measure the mice weight and tumor perpendicular diameters every 3 days. The tumor volume (*V*) was estimated as follows:

$$ V=L\times {W}^2/2 $$

where *L* and *W* are the two perpendicular diameters.

Mice were sacrificed 15 days after the first treatment. Tumors were resected and prepared for further flow cytometry analysis.

### Statistical analysis

Representative measurement data from three independent experiments were presented as mean ± SEM. For non-repeated measurement data, two-tailed Student’s *t* test was used for comparison between the two groups. For repeated measurement data, repeated measures analysis of variance was used. *p* < 0.05 was considered significant. Linear regression was performed to study the possible correlation between enrichment of H3K9Ac and B7-H1 transcription level. GraphPad Prism version 6.0 was used to perform the statistical analyses.

## Results

### B7-H1 expression was correlated with HDAC expression in GC

The mammalian HDAC family comprises 18 members, which are grouped into 4 classes, based on their homology to yeast deacetylase proteins [35, 36]. Class I HDACs are similar to yeast Rpd3, consisting of HDAC1, 2, 3, and 8, and are expressed ubiquitously and located mainly in the nucleus. Class II HDACs are related to yeast Had1, consisting of HDAC4, 5, 6, 7, 9, and 10, and can shuttle between cytosol and nucleus. Class III HDACs are homologs to yeast Sir2, requiring NAD+ for their activity, including SIRT1–7. HDAC11 is the only member of class IV HDAC, sharing homology with both Rpd3 and Hda1.

**Fig. 1:**
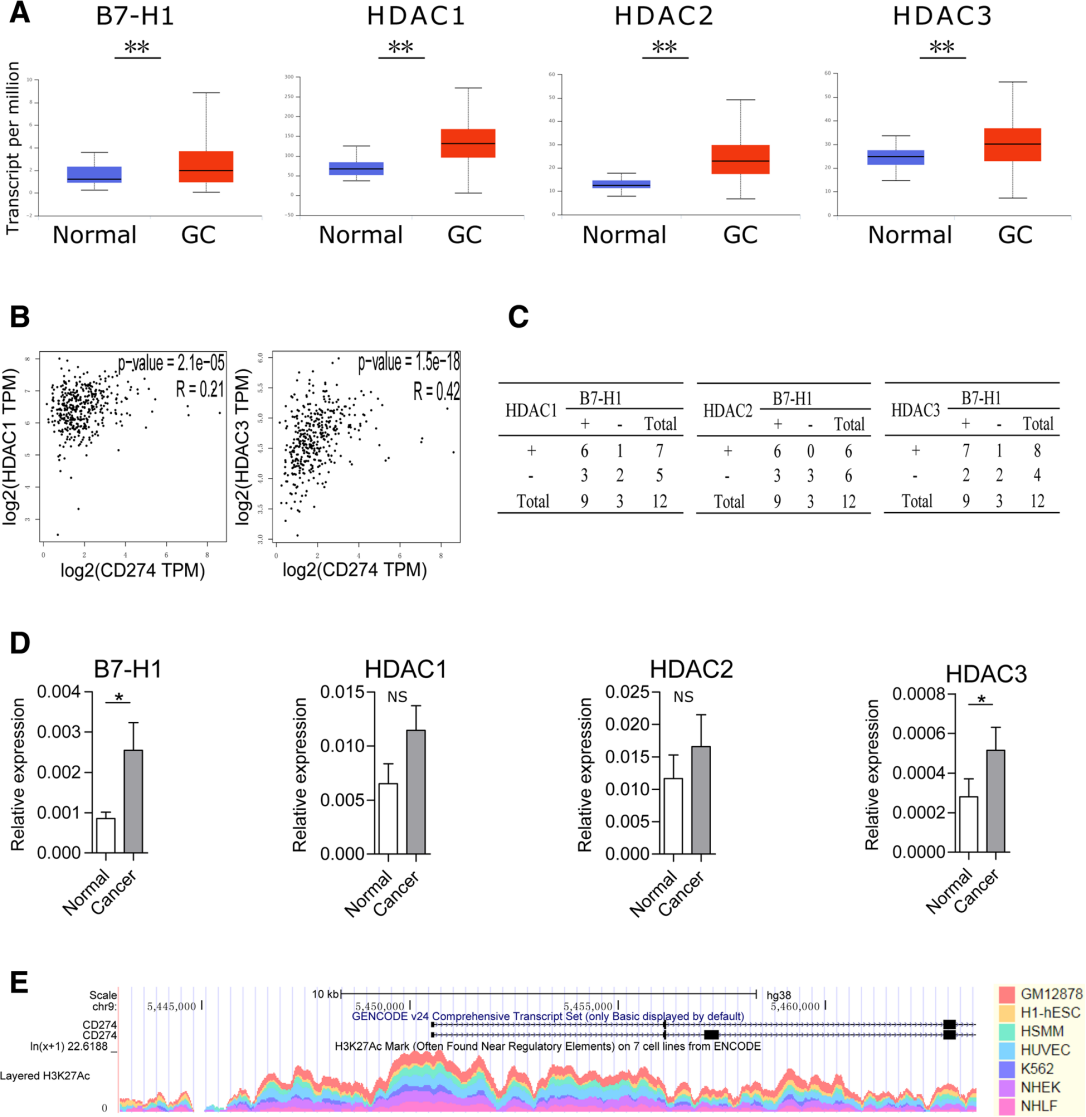
B7-H1 expression is correlated with HDAC in GC. **a** B7-H1 and HDAC1–3 expression was compared between normal gastric tissues (*n* = 34) and primary GC tissues (*n* = 415) from RNA-seq data of TCGA. The results were presented in box-and-whisker plots (median values (line), 25th–75th percentiles (box outline) and minimum and maximum values (whiskers)). **b** Spearman correlation analyses of B7-H1 and HDACs were performed using GC RNA-seq data of TCGA. **c** B7-H1 and HDAC1–3 protein expression was compared between 12 paired GC tissues and cancer-adjacent tissues collected from our institution. Shown were expression change between each pair of cancer and adjacent tissue. +, expression upregulation in cancer; −, expression downregulation in cancer. **d** B7-H1 and HDAC1–3 mRNA expression was compared between 12 paired GC tissues (cancer) and tumor-adjacent tissues (normal) collected from our institution. **e** H3K27Ac level in B7-H1 gene promoter region was assessed in seven cell lines using ChIP-seq data from ENCODE. *p < 0.05, **p < 0.001. NS, not significant

We mined public available database TCGA to examine the differential expression of B7-H1 and HDAC between normal and GC tissues. Analysis of RNA-seq data from 34 normal gastric tissues and 415 primary GC revealed significant upregulation of B7-H1; HDAC1–3, 6–8, and 10; and SIRT1, 3, 5, and 6 and downregulation of HDAC5 and SIRT4 in cancer (Fig. 1a, only data of B7-H1 and HDAC1–3 was presented). Correlation analysis from TCGA data revealed that HDAC3 and HDAC1 were significantly correlated with B7-H1 expression in GC with a respective r value of 0.42 (*p* < 0.001) and 0.21 (*p* < 0.001) (Fig. 1b). The results suggested that HDACs, especially HDAC1–3, may have a potential role in B7-H1 regulation. Furthermore, we determined the mRNA and protein expression levels of B7-H1 and HDAC1–3 in 12 GC and adjacent tissues collected from our institution. Western blot results showed that HDAC1, HDAC2, and HDAC3 expression change between cancer, and adjacent tissue was consistent with the B7-H1 level change in 8 (66.7%), 9 (75.0%), and 9 (75.0%) cases, respectively (Fig. 1c, Additional file 1: Figure S1). RT-qPCR results showed that B7-H1 and HDAC3 were significantly upregulated in GC tissues, while HDAC1 and HDAC2 upregulation did not reach statistical significance (Fig. 1d). Acetylation of histone H3 lysine 27 (H3K27Ac) in the gene promoter region is a marker of active gene transcription [37]. Chip-seq data from ENCODE showed that on GM12878, H1-hESC, HSMM, HUVEC, K562, NHEK, and NHLF cell lines, the B7-H1 gene promoter region was abundant with H3K27Ac mark, linking histone acetylation directly to the B7-H1 gene regulation (Fig. 1e). Overall, combined bioinformatics and clinical sample data indicated HDACs, especially HDAC1–3 may have a potential role in B7-H1 expression regulation in GC.

### HDAC inhibition reduced IFN-γ-induced B7-H1 expression in GC

**Fig. 2:**
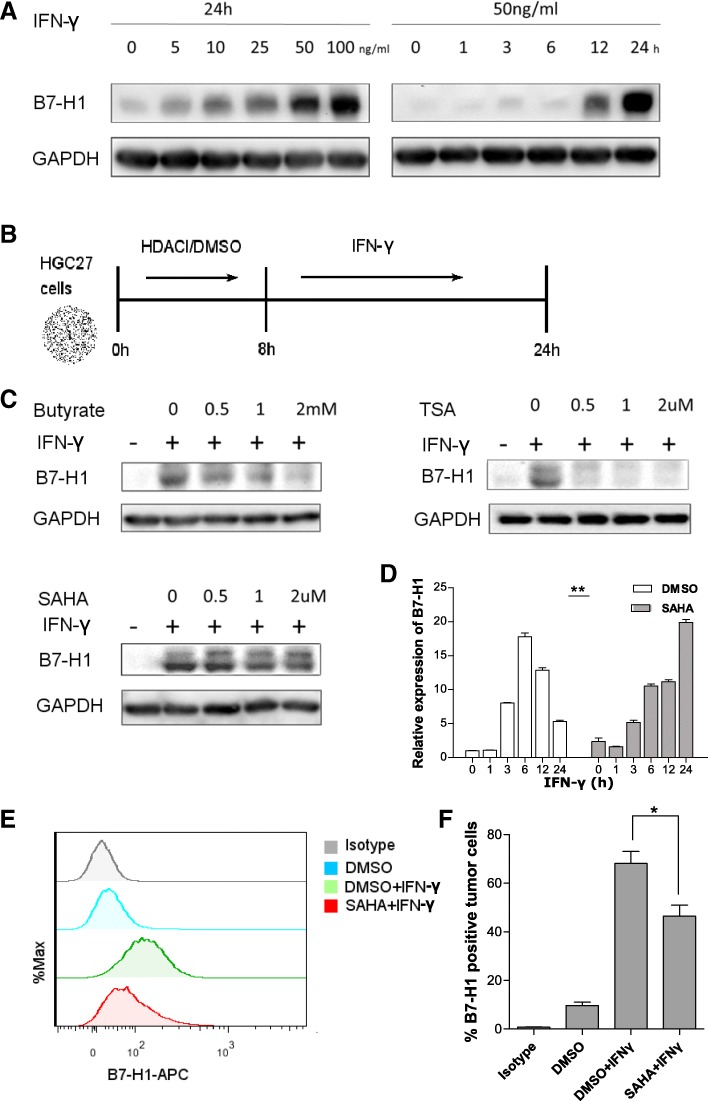
HDAC inhibition suppressed IFN-γ-induced B7-H1 expression in GC. **a** HGC27 cells were treated with IFN-γ of 0 to 100 ng/ml for 24 h or of 50 ng/ml for 0 to 24 h before B7-H1 protein expression was determined. **b** As was shown in the treatment schedule, HGC27 cells were pretreated with either DMSO or different concentrations of HDACI (sodium butyrate, TSA, or SAHA) for 8 h, which was then removed, followed by treatment with 50 ng/ml IFN-γ for 24 h. **c** HGC27 cells were treated according to the above schedule; then, B7-H1 protein expression was determined. **d** HGC27 cells were treated according to the above schedule; then, B7-H1 mRNA expression was determined. **e**, **f** HGC27 cells were treated according to the above schedule; then, cell surface B7-H1 expression was determined by flow cytometry. **c**–**e** Experiments were repeated three times with similar results. *p < 0.05, **p < 0.001

Next, we wondered whether HDAC inhibition had an effect on IFN-γ-induced B7-H1 expression in GC. Firstly, we confirmed that IFN-γ induced strong expression of B7-H1 on GC cells. Under rest state, HGC27 cells showed little or no expression of B7-H1. After treatment with IFN-γ of different concentration for varying length of time, HGC27 cells showed concentration and time-dependent upregulation of B7-H1, reaching the peak expression level with 100 ng/ml IFN-γ stimulation for 24 h (Fig. 2a). We next investigated HDACI’s effect on the B7-H1 induction. Sodium butyrate, TSA, and SAHA are HDACIs with different molecular structures. Pretreatment with HDACI significantly reduced IFN-γ-induced B7-H1 expression (Fig. 2b). To investigate the effect at the transcription level, RT-qPCR was performed (Fig. 2c). The results showed that SAHA treatment alone was able to upregulate basal B7-H1 mRNA expression by about twofolds. However, IFN-γ-induced B7-H1 expression was reduced at 3, 6, and 12 h post-stimulation. Furthermore, the kinetics of B7-H1 mRNA induction was altered. SAHA pretreatment caused a delay of the peak B7-H1 mRNA level from 6 to 24 h after IFN-γ stimulation. As SAHA was only applied for 8 h and then removed prior to IFN-γ stimulation, the altered kinetics of B7-H1 mRNA expression was likely the result of HDAC activity returning. Cell surface expression of B7-H1 is important for its function. Flow cytometry confirmed that cell surface expression of B7-H1 was also reduced (Fig. 2d, e).

### HDAC knockdown suppressed IFN-γ-induced B7-H1 expression in GC

**Fig. 3:**
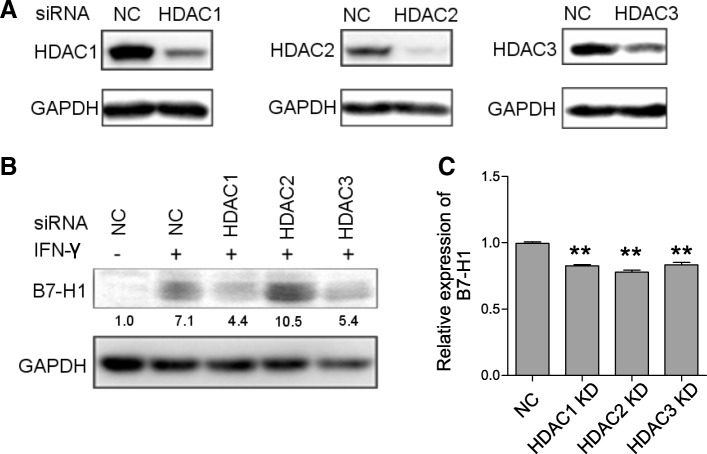
HDAC knockdown suppressed IFN-γ-induced B7-H1 expression in GC. **a** 36 h after transfection of negative control (NC) or HDAC siRNA, HDAC1, HDAC2, and HDAC3 protein expression in HGC27 cells was determined, respectively. **b** Following siRNA transfection, HGC27 cells were treated with 50 ng/ml IFN-γ for 24 h. Then, B7-H1 protein expression was determined. **c** Following siRNA transfection, HGC27 cells were treated with 50 ng/ml IFN-γ for 12 h. Then, B7-H1 mRNA expression were determined. **a**–**c** Experiments were repeated three times with similar results. **p < 0.001

One of the limitations of HDACI used above is that they are not HDAC subclass-specific. To linearize specific HDAC’s role in IFN-γ-induced B7-H1 expression, we performed HDAC gene knockdown. Based on the bioinformatics study, we focused on HDAC1–3. siRNA transfection effectively downregulated the expression of HDAC1–3. Following the knockdown, HGC27 cells were stimulated with IFN-γ for 24 h, and B7-H1 expression was determined. Western blot showed that HDAC1 and HDAC3 knockdown significantly reduced induced B7-H1 expression compared to negative control siRNA transfection, while HDAC2 knockdown had no such effect (Fig. 3a, b). RT-qPCR revealed that HDAC1–3 knockdown all reduced induced B7-H1 mRNA expression (Fig. 3c). The results suggested HDAC1 and HDAC3 had specific roles in regulating IFN-γ-induced B7-H1 in GC.

### SAHA pretreatment or HDAC knockdown impaired early phase IFN-γ signaling through JAK2 downregulation

**Fig. 4:**
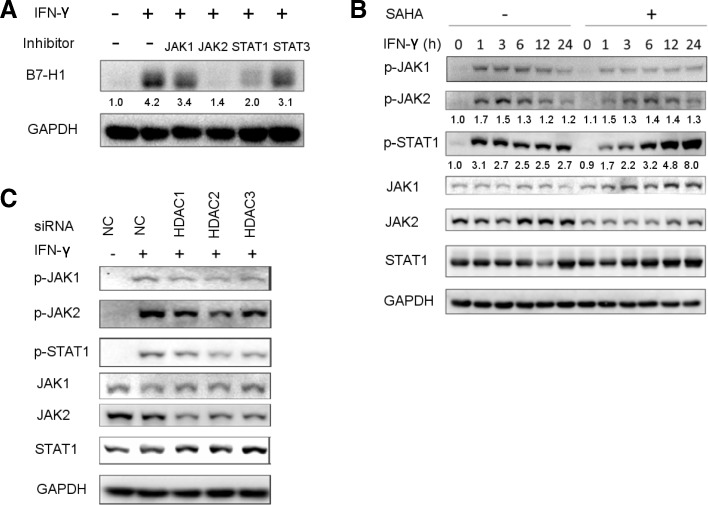
SAHA pretreatment or HDAC knockdown suppressed the expression of p-JAK1, p-JAK2, and p-STAT1. **a** HGC27 cells were pretreated with either DMSO or GLPG0634 (5 μM), fedratinib (5 μM), fludarabine (100 μM), and NSC 74589 (200 μM) for 8 h to inhibit JAK1, JAK2, STAT1, and STAT3, respectively, followed by treatment of 50 ng/ml IFN-γ for 24 h. Then, B7-H1 protein expression was determined. **b** HGC27 cells were treated according to the schedule shown in Fig. 2b; then, the expression of the phosphorylated and total protein of JAK1, JAK2, and STAT1 was determined. **c** Thirty-six hours after the transfection of negative control (NC) or HDAC siRNA, HGC27 cells were treated with 50 ng/ml IFN-γ for 3 h. Then, the expression of the phosphorylated and total protein of JAK1, JAK2, and STAT1 was determined. **a**–**c** Experiments were repeated three times with similar results

**Fig. 5:**
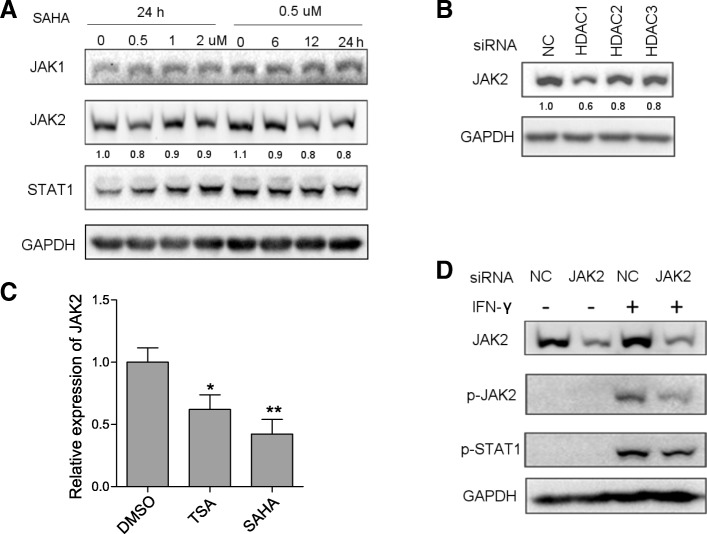
HDAC inhibition or HDAC knockdown suppressed JAK2 expression. **a** HGC27 cells were treated with 0–2 μM SAHA for 24 h or with 0.5 μM SAHA for 0–24 h prior to JAK2 protein expression determination. **b** Thirty-six hours after transfection of NC or HDAC siRNA, JAK2 protein expression in HGC27 cells was determined. **c** Twenty-four hours after DMSO, SAHA (0.5 μM), or TSA (0.5 μM) treatment, JAK2 mRNA expression in HGC27 cells was determined. **d** Thirty-six hours after transfection of NC or JAK2 siRNA, HGC27 cells were treated with 50 ng/ml IFN-γ for 3 h. Then JAK2, p-JAK2, and p-STAT1 protein expression were determined. **a**–**d** Experiments were repeated three times with similar results. *p < 0.05, **p < 0.001

Since IFN-γ-induced gene expression relies on robust signaling transduction, we investigated HDACI’s impact on IFN-γ signaling pathway. The key components of the pathway include JAK1, JAK2, and STAT1, all of which are tyrosine kinases and can be activated through tyrosine phosphorylation. Upon IFN-γ binding to the interferon gamma receptor (IFNGR), JAK1 and JAK2 phosphorylate each other and then they phosphorylate IFNGR1 to allow docking and phosphorylation of STAT1 [38]. Inhibition of JAK2 and STAT1 resulted in reduced IFN-γ-induced B7-H1 expression in HGC27 cells, while a minor effect was observed for JAK1 and STAT3 inhibition (Fig. 4a). Among them, JAK2 inhibition had the strongest effect, almost completely abrogating IFN-γ-induced B7-H1 expression. We further investigated the effects of HDACI on JAK1, JAK2, and STAT1 expression and phosphorylation. SAHA pretreatment significantly reduced the expression level p-JAK1, p-JAK2, and p-STAT1 at 1 h and 3 h post-IFN-γ stimulation (Fig. 4b). Normally, IFN-γ-induced signaling would eventually resolute, and the expression of phosphorylated proteins went back to the pre-stimulation level, which started to happen at 12 h post-stimulation in HGC27 according to our results. Interestingly, SAHA pretreatment caused the persistent relatively high level of p-JAK1, p-JAK2, and p-STAT1 at 12 h and 24 h post-IFN-γ stimulation. The data indicated that SAHA impaired early phase IFN-γ signaling, although it seemed to enhance prolonged signaling. Next, we performed HDAC knockdown followed by IFN-γ stimulation. HDAC1–3 knockdown also reduced the expression of p-JAK1, p-JAK2, and p-STAT1 at 3 h after IFN-γ stimulation (Fig. 4c). Noticeably, SAHA pretreatment and HDAC knockdown reduced JAK2 expression while JAK1 and STAT1 were not affected. To further validate the results, we treated HGAC27 cells with varying concentration of SAHA for a different length of time. We found that SAHA downregulated the JAK2 expression at different concentration and time (Fig. 5a). HDAC, especially HDAC1 knockdown, had the same effect (Fig. 5b). RT-qPCR showed that both TSA and SAHA could downregulate JAK2 mRNA level (Fig. 5c). In light of JAK2’s key role in IFN-γ signaling and its upstream position in the pathway, we hypothesized that JAK2 downregulation was the mechanism behind SAHA or HDAC knockdown’s effect. To support this, JAK2 knockdown downregulated p-JAK2 and p-STAT1 level following IFN-γ stimulation (Fig. 5d). Overall, the results suggested that impairment of IFN-γ signaling caused by HDAC inhibition was mediated by JAK2 downregulation.

### SAHA pretreatment impaired STAT1 nuclear translocation

**Fig. 6:**
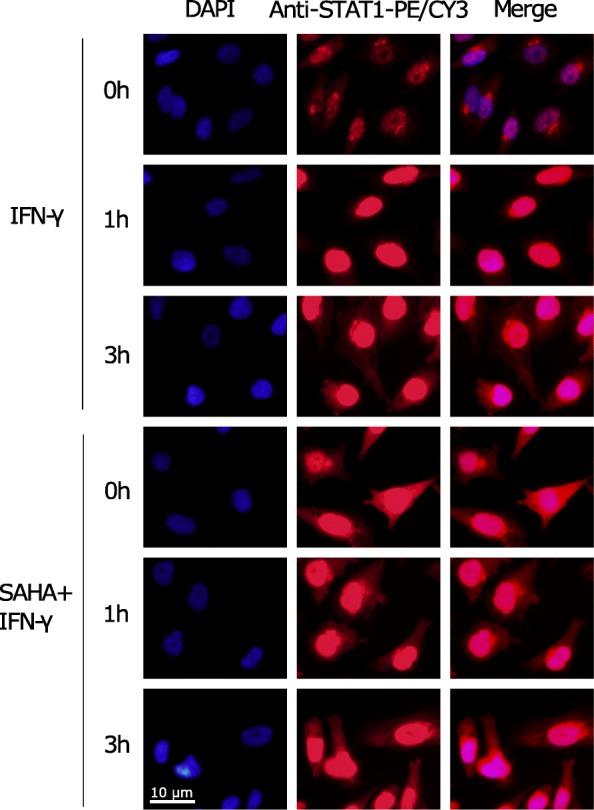
SAHA pretreatment impaired STAT1 nuclear translocation. HGC27 cells were pretreated with either DMSO or 2 μM SAHA for 8 h before stimulation with 50 ng/ml IFN-γ for 1 h or 3 h. Immunofluorescence staining of STAT1 was performed. The experiment was repeated three times with similar results

Nuclear translocation of STAT1 is an important step of the IFN-γ signal transduction. Following phosphorylation, STAT1 homodimerized and was translocated from the cytoplasm to the nucleus [39]. We investigated the effects of SAHA on the process (Fig. 6). Immunofluorescence staining 1 h and 3 h after IFN-γ stimulation showed that IFN-γ induced efficient STAT1 translocation. However, SAHA pretreatment significantly enhanced the cytoplasm intensity of STAT1, which indicates cytoplasm retention. The results indicated that SAHA impaired IFN-γ-induced STAT1 nuclear translocation.

### SAHA pretreatment impaired IFN-γ-induced histone acetylation of B7-H1 gene promoter

**Fig. 7:**
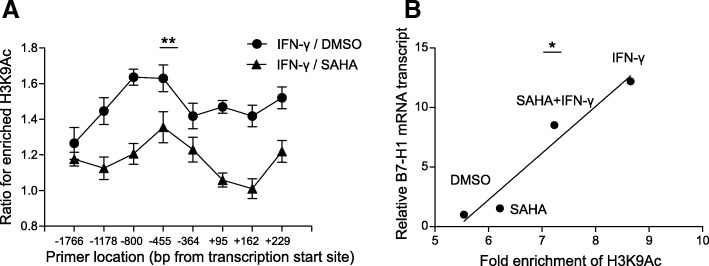
SAHA pretreatment impaired IFN-γ-induced histone acetylation of B7-H1 gene promoter. **a** HGC27 cells were pretreated with either DMSO or 2 μM SAHA for 8 h before stimulation with 50 ng/ml IFN-γ for 3 h. ChIP was performed using anti-H3K9Ac followed by quantification of immunoprecipitated B7-H1 gene promoter through PCR. H3K9Ac enrichment was determined by the percent input method. IFN-γ-induced upregulation of H3K9Ac was calculated as the ratio of H3K9Ac enrichment at post-stimulation to that at pre-stimulation. **b** The correlative relationship between enrichment of H3K9Ac at the promoters of B7-H1 gene and relative B7-H1 mRNA transcription in the same cell populations mentioned in A. *X*-axis stood for the mean value of enrichment of H3K9Ac at all the promoters of B7-H1 gene. *Y*-axis stood for B7-H1 mRNA expression relative to pre-stimulation level. **a**, **b** Experiments were repeated three times. **p < 0.001, *p < 0.05

Acetylation of histone and subsequent chromatin remodeling is one of the integral parts for IFN-γ-induced gene transcription [40–42]. STAT1 binding to the GAS sequence recruited HAT and HDAC to induce histone hyperacetylation [43, 44]. We investigated the effect of HADC inhibition on IFN-γ-induced histone acetylation on B7-H1 gene promoter region through ChIP-qPCR assay. Enrichment of H3K9Ac, a marker of active transcription, was upregulated by IFN-γ up to 1.6-folds at − 455 bp from the TSS (Fig. 7a). However, following SAHA pretreatment, IFN-γ only induced an upregulation of 1.3-folds at the same site. The same trend was observed for other sites along the B7-H1 gene regulatory region, ranging from − 1766 to + 229 bp from TSS. Furthermore, we showed that the enrichment of H3K9Ac in the promoter area significantly correlated with B7-H1 level with a *r*^2^ of 0.94 (Fig. 7b). The data showed that IFN-γ-induced histone acetylation of B7-H1 gene promoter was inhibited by SAHA.

### SAHA inhibited tumor growth and reduced tumor B7-H1 expression in subcutaneously transplanted mouse GC model

**Fig. 8:**
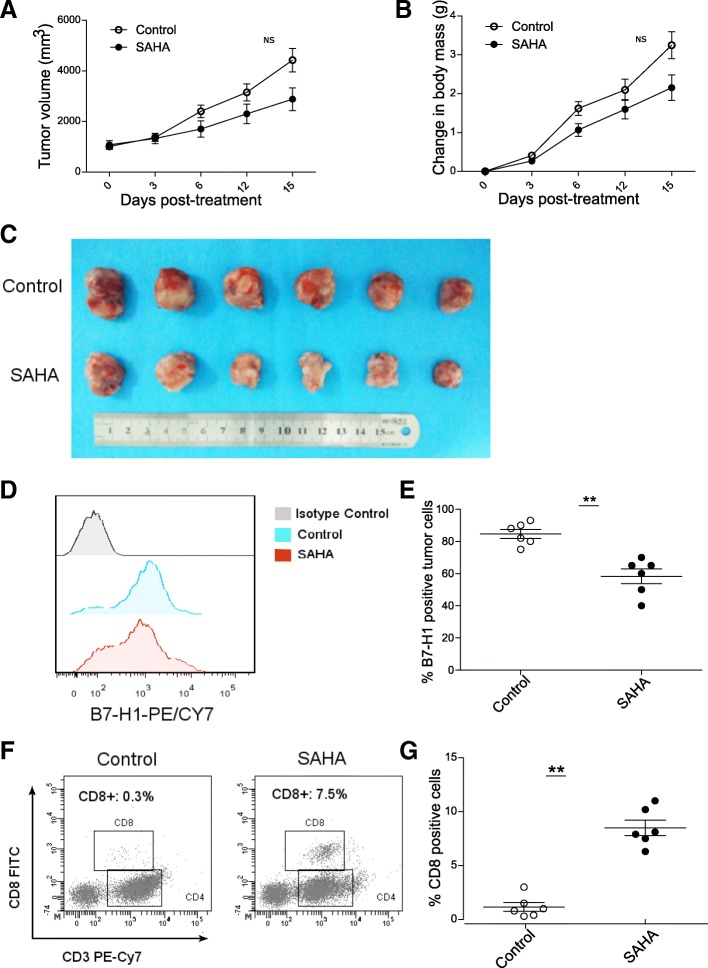
SAHA inhibited tumor growth and suppressed tumor B7-H1 expression in subcutaneously transplanted mouse GC model. **a**–**c** SAHA treatment group showed slower tumor growth and smaller tumor size compared to control group. **d**, **e** Tumor cell B7-H1 expression was lower in SAHA group than in the control group. **f**, **g** The percentage of tumor infiltrated CD8+ tumor cells was higher in the SAHA group than in the control group. Shown in **d** and **f** are representative results in one mouse. NS, not significant; **p < 0.001

We investigated HDACI’s effect on tumor growth and B7-H1 expression in vivo using the subcutaneously transplanted mouse GC model. A trend of slower tumor growth and smaller tumor size in SAHA group compared to that of the control group was observed (Fig. 8a–c). However, the difference did not reach statistical significance. The percentage of B7-H1-positive tumor cells was also lower in SAHA group (Fig. 8d, e). Considering B7-H1’s role in regulating tumor immunity, we further determined the amount of tumor-infiltrating CD8+ T cells (Fig. 8f, g). A higher percentage of infiltrating CD8+ T cells was observed in tumors in SAHA group than those in the control group.

## Discussion

Our study revealed that HDAC is indispensable for the expression of B7-H1 in GC, as HDAC inhibition reduced IFN-γ-induced B7-H1 expression. HDAC has long been deemed as an eligible target in cancer [23]. B7-H1 is the recently defined immune checkpoint, and B7-H1 blockade using monoclonal antibodies showed great potential as a new treatment modality [10]. Thus, our study suggested that HDAC may dictate how robustly cells will respond to the IFN-γ in the tumor microenvironment, raising the possibility of targeting B7-H1 using HDAC inhibitors.

Our results revealed that HDACI impaired early phase IFN-γ signaling by downregulating JAK2. JAK2 is the key upstream component of the IFN-γ signaling pathway, the absence of which led to unresponsiveness to IFN-γ [38, 45, 46]. However, the previous studies reported several distinct mechanisms behind HDACI-elicited impaired IFN-γ signaling. One proposed that HDACI caused STAT1 hyperacetylation, which compromised its phosphorylation and activation [47, 48]. But the results were unable to be repeated by another group [49]. Others reported that HDACI upregulated suppressor of cytokine signaling (SOCS) gene expression, which is responsible for negative feedback regulation of IFN-γ signaling pathway [50, 51]. SOCS can downregulate JAK2 by accelerating its degeneration [52]. We revealed that both JAK2 mRNA and protein expression were decreased by HDACI treatment, which indicated HDAC’s role in transcriptional regulation of JAK2. Our results suggested certain HDACs took part in regulating B7-H1 expression. Recently isoform-selective HDAC inhibitors have been developed [53–55]. Whether they have a role in the more specific and targeted inhibition of B7-H1 than pan-HDAC inhibitors such as SAHA and TSA remains to be determined.

HDACI has been shown to have widespread immuno-regulatory effects. Some reported that HDACI enhanced tumor immunity. It upregulated major histocompatibility complex (MHC) and co-stimulators CD40 and CD86 expression, promoting melanoma cells’ antigen presenting capability [24]. It suppressed regulatory T cells by modulating forkhead box P3 (Foxp3) expression [25, 26]. Furthermore, it upregulated the expression of natural killer group 2 member D (NKG2D) ligands on tumor cells, enhancing NK cell-mediated killing [56]. In contrast, others reported that HADCi repressed tumor immunity. For example, it increased myeloid-derived suppressor cell (MDSC) production [57]. The contradicting conclusions may derive from distinct experiment models and systems involved. From our point of view, HDACI enhanced tumor immunity by suppressing tumor B7-H1 expression in vitro and in vivo. The immuno-regulatory effect makes HDACI exert anti-tumor function independent of its cytotoxic effects. That makes it the ideal candidate for combinatory therapy with anti-B7-H1 to enhance efficacy and limit adverse effects. As a matter of fact, clinical trials evaluating safety and efficacy of combinatory therapy of HDACI and anti-B7-H1 in cancer patients are already on the way [58].

In our study, the observed in vivo tumor inhibition effect of HDACI was modest. There are three possible reasons. One is that the HDACI at the dose we used did not inhibit B7-H1 expression to an optimal level. As was shown in FACS results, relatively high expression of B7-H1 remained on tumors cells. The second is that compared with cell toxicity effects, immunological anti-tumor effects tend to require a longer time to show. In the short observation period, as was used in our study, immunological effects exerted by HDACI probably did not reach its maximal level. Thirdly, gastric cancer is a multifactorial and aggressive tumor. In our study, we focused on the possible immunological aspect of the tumor, while other factors may also have roles in promoting its growth. From our results, we inferred that the immunological effect may play a relatively minor part.

There are several limitations and unaddressed questions in our study. Firstly, HDACI’s inhibition of B7-H1 may be accompanied by other genes’ expression change because of the repression of the entire IFN-γ signaling. Secondly, the effect may be cell type specific. Thirdly, pan-HDACIs were used, and HDAC subtype’s function still needed to be further linearized. Besides, HDACI’s effect on the B7-H1 expression on the tumor-infiltrating immune cell was not examined.

## Conclusions

IFN-γ-induced B7-H1 expression in GC cells requires HDAC. Inhibition of HDAC impaired IFN-γ’s ability to induce B7-H1 expression, resulting in reduced tumor growth and increased tumor-infiltrating CD8-positive T cells in mouse subcutaneous model. Mechanistically, inhibition of certain HDAC downregulated the expression of JAK2, the key component of the IFN-γ signaling pathway, which subsequently led to compromised activation of IFN-γ signaling and histone acetylation in B7-H1 gene promoter region. The role of HDAC in the regulation of B7-H1 expression suggests small molecular HDACI as a potential way of targeting B7-H1 in GC.

## Additional file

Additional file 1: **Figure S1.** HDAC1–3 expression correlated B7-H1 expression in clinical gastric specimens. (A) B7-H1 and HDAC1–3 expression in 12 paired gastric cancer (C) and adjacent tissues (A) were determined by western blot. (B) After quantification and normalization, the expression change of HDAC1–3 and B7-H1 between each pair of cancer and adjacent tissue was presented. A, the experiment was repeated three times with similar results. (PDF 105 kb)

## Abbreviations

ChIP: Chromatin immunoprecipitation
HDAC: Histone deacetylase
JAK1: Janus kinase 1
STAT1: Signal transducer and activator of transcription 1
