# Supplementary figures and images for: HDAC is indispensable for IFN-γ-induced B7-H1 expression in gastric cancer
Source: Clin Epigenetics. 2018 Dec 11;10:153. doi: 10.1186/s13148-018-0589-6 (PMC6288935; doi:10.1186/s13148-018-0589-6)

**A**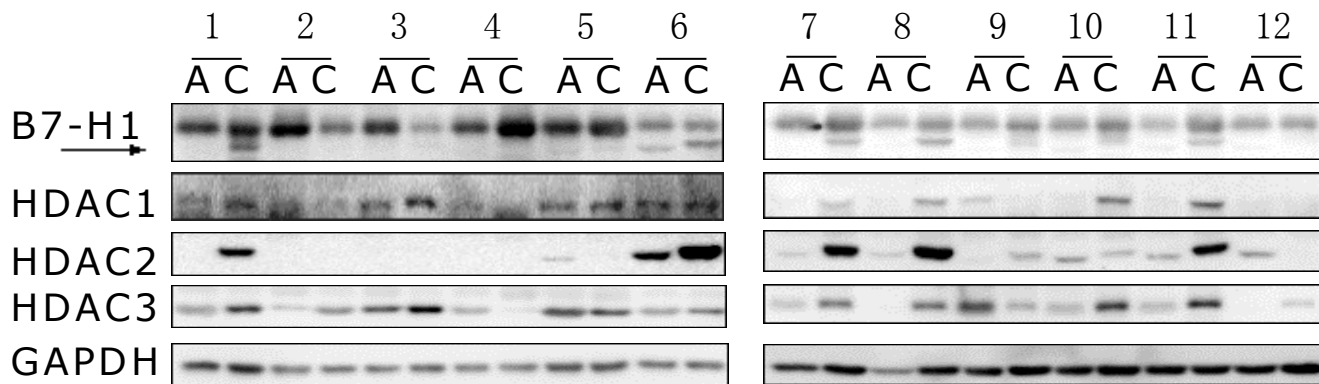**B**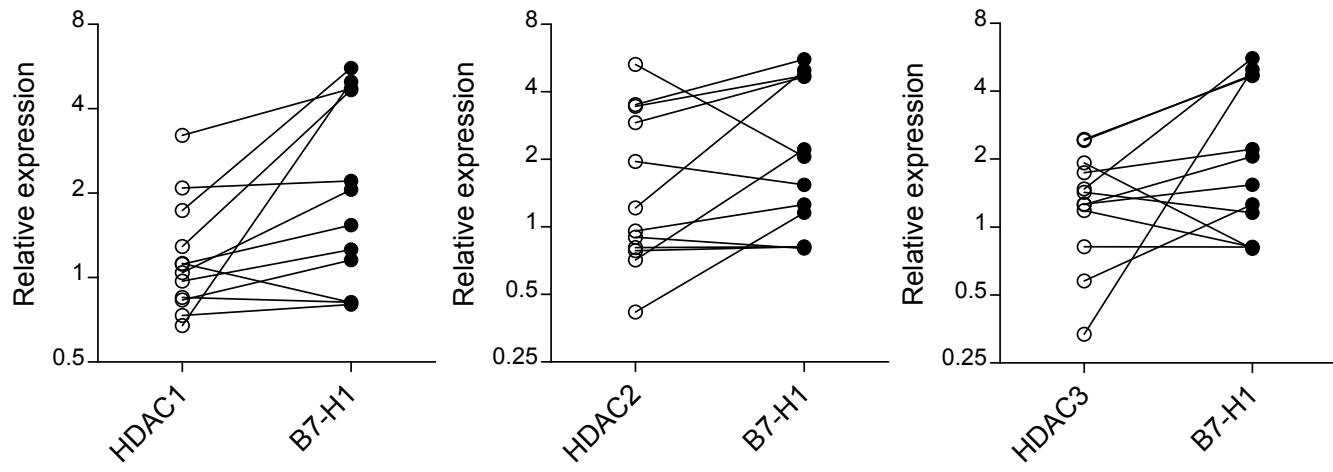

Supplement: Supplementary file 1 — Figure S1. HDAC1–3 expression correlated B7-H1 expression in clinical gastric specimens. (A) B7-H1 and HDAC1–3 expression in 12 paired gastric cancer (C) and adjacent tissues (A) were determined by western blot. (B) After quantification and normalization, the expression change of HDAC1–3 and B7-H1 between each pair of cancer and adjacent tissue was presented. A, the experiment was repeated three times with similar results. (PDF 105 kb) [file 13148_2018_589_MOESM1_ESM.pdf]
